# Dementia with Lewy bodies versus nonconvulsive status epilepticus in the diagnosis of a patient with cognitive dysfunction, complex visual hallucinations and periodic abnormal waves in EEG: a case report

**DOI:** 10.1186/1471-2377-14-112

**Published:** 2014-05-22

**Authors:** Li Sun, Jie Cao, Feng na Chu, Zan Wang, Yudan Lv

**Affiliations:** 1 Department of Neurology, The First hospital of JiLin University, 71-XinminStreet, ChangChun, P. R. China

**Keywords:** Visual hallucinations, Periodic synchronous discharges (PSD), Nonconvulsive status epilepticus (NCSE), Dementia with Lewy bodies (DLB)

## Abstract

**Background:**

The diagnosis of dementia with Lewy bodies (DLB) is often challenging in elderly individuals, not only for its various clinical features, sometimes, but also for its rare changes of periodic synchronous discharges (PSD) in electroencephalogram ( EEG). So, we reported one case of DLB and gave a detailed analysis.

**Case presentation:**

A Chinese patient (Female, 56 years old) presented with progressive cognitive decline and complex visual hallucinations. Several days after admission, she gradually showed focal myoclonic jerks. Mini-Mental State Examination (MMSE) score was 19/30, EEG revealed PSD, Cerebrospinal fluid and 14-3-3 brain protein was negative, Magnetic resonance imaging (MRI) showed diffuse atrophy. To differentiate the PSD derived from DLB or from late-onset Absence Status Epilepticus, we have given the treatment with intravenous valproate (1200 mg/24 h) and diazepam 20 mg under the EEG monitor, a clinical improvement was absent and PSD in EEG did not disappear. Two weeks later, the patient showed akinetic-rigid syndrome and PSD in EEG persisted for a long time. According to her history and therapy, a clinical diagnosis of DLB has been made, but no autopsy for confirmation, and in the following visit, she has a poor prognosis.

**Conclusion:**

PSD in EEG may occasionally be recorded in neurodegenerative disorders such as AD, DLB other than CJD or NCSE. Hence it should not dissuade clinicians from the diagnosis of DLB where the clinical and neuropsychological findings were consistent with suggested diagnostic criteria for DLB.

## Background

The diagnosis of dementia with Lewy bodies (DLB) is often challenging in elderly individuals because various symptoms of this condition overlap with other conditions that are common in this population, such as Alzheimer’s disease (AD), Creutzfeldt-Jakob disease (CJD) [1] or Nonconvulsive status epilepticus (NCSE). However, DLB also has a higher mortality and morbidity just as AD or CJD. Life expectancy in DLB is about 5–8 years [2] Thus, a comprehensive understanding of DLB is very critical, not only for its various clinical features, but also for its examination, especially the electroencephalogram ( EEG).

The EEG has been considered as a valuable neurophysiological method in the evaluation of the dementia so far. It is well-known that the existence of PSD in a patient with progressive dementia is rather suggestive of CJD. Nevertheless, the periodic abnormalities have been rarely described in others types of dementia such as AD, DLB [3-7], and have also been rarely described in the elderly men with NCSE such as late-onset Absence Status Epilepticus.

In this case, we reported one case of DLB with progressive cognitive impairment, intermittent,vivid, complex visual hallucinations,focal myoclonic jerks, and akinetic-rigid syndrome, then gave a detailed analysis of the EEG changes. The patient’s husband has provided written informed consent and this case report has been approved by our hospital’s Research Ethics Board.

## Case presentation

A Chinese 56-year-old female patient presented with progressive cognitive decline accompanied with dizziness for one month, her family members told us that she was always unable to remember somethings happened recently and was occasionally unable to find home. A week later, she gradually showed complex visual hallucinations, the visual hallucinations were stereotypical: somebody was jumping, singing, dancing or talking with her. The visual hallucinations persisted for several minutes and occurred dozens of times each day. At the same time, the patient was frightened. So she was admitted to our hospital by her family members, however, after admission, she gradually showed focal myoclonic jerks.

**Figure 1:**
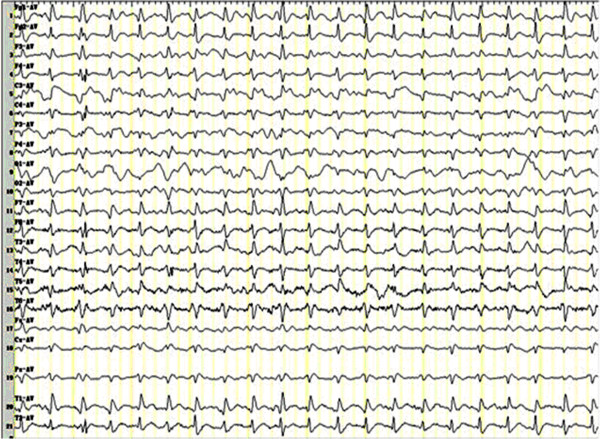
Generalized periodic synchronous discharges (PSD) in EEG.

On admission, the physical examination was unremarkable. A Mini-Mental State Examination (MMSE) score was 19/30, revealed that she had trouble in recalling words and locating. Comprehensive neuropsychological testing was performed, such as the Chinese Revised Wechsler Adult Intelligence Scale (WAIS-RC) and the Chinese Revised Wechsler Memory Scale (WMS). She had a lower performance IQ than verbal IQ, which may indicated the impaired function of nondominant hemisphere associated with visuospatial function, especially poor performance on visual recognition, visual regeneration and picture recall, such as Table 1. Her EEG was performed immediately to evaluate her frequent visual symptoms and focal myoclonic jerks. We found that her EEG was dominated by the presence of generalized periodic synchronous discharges (PSD) every 0.5 to 1.0 seconds (Figure 1), cerebrospinal fluid revealed the normal values for protein and cells, the determination of 14-3-3 brain protein was negative. Magnetic resonance imaging of the brain showed diffuse atrophy.

**Table 1:** WAIS-RC and WMS test results

	**WAIS-RC**	**Score**	**WMS-RC**	**Score**		
VIQ	Information	9.3 (0–27)	Associative memory	9.9 (0–21)		
	Comprehension	10.2 (0–27)	Tactile memory	9.6 (0–27)		
	Arithmetic	10.2 (0–18)	Comprehension memory	10.1 (0–25)		
	Similarities	8.6 (0–26)	Digit span	11.4 (0–20)		
	Vocabulary	8.4 (0–80)	Number sequence	9.1 (0–51)		
	Digit span	12.1 (0–76)				
PIQ	Symbol search	8.9 (0–21)	Location	4.6 (0–5)		
	Block design	8.6 (0–48)	Visual recognition	8.9 (0–16)		
	Picture concept	7.2 (0–37)	Visual regeneration	7.4 (0–14)		
	Picture completion	7.3 (0–42)	Picture recal	8.9 (0–20)		

After the EEG monitoring, two diseases have been taken into account: DLB or NCSE such as late-onset elderly Absence Status Epilepticus. In order to differentiate the PSD derived from DLB or from late-onset Absence Status Epilepticus, we have given her intravenous valproate (1200 mg/24 h) and diazepam (20 mg), however, a clinical improvement was absent and the PSD in EEG didn’t disappear. Two weeks later, the patient showed akinetic-rigid syndrome and PSD in EEG persisted for a long time. Combing her detailed history with the therapy, we have made a clinical diagnosis of DLB according to the clinical criteria [8,9]: central feature such as progressive dementia, core features such as fluctuating impaired cognition ,visual hallucinations, akinetic-rigid syndrome. In our report, the patient has a history of progressive dementia for two months accompanied with fluctuating impaired cognition such as that she was occasionally unable to find home or remember somethings, and showed complex visual hallucinations and akinetic-rigid syndrome plus myoclonus, which were consistent with the clinical diagnosis criteria. The patient’s family members refused the autopsy for confirmation and the therapy. In the following visit, the patient had difficulty in eating, developed acute pancreatitis, and will have a poor prognosis.

## Conclusions

Cognitive impairment punctuated by complex vivid visual hallucinations, was our patient’s main complaint, which can be seen in DLB or Nonconvulsive status epilepticus, but soon after admission, she gradually showed focal myoclonus and akinetic-rigid syndrome, which would be helpful in the clinical diagnosis of DLB. DLB was recently defined and revised as a separate disease entity according to Mckeith et al. [9], included core features such as fluctuation cognition, visual hallucinations, spontaneous parkinsonism, if two core features are presented, it will be sufficient for a diagnosis of DLB. Besides this, if any core features is absence, one or more suggestive features will be sufficient for possible DLB, such as REM sleep behavior disorder, severe neuroleptic sensitivity, low dopamine transporter uptake in basal ganglia demonstrated by SPECT or PET imaging. However, supportive features are commonly presented in DLB but lack of sufficient diagnostic specificity, such as prominent slow wave activity in EEG with temporal transient sharp waves, which may be presented in the intermediate phase of DLB. It is well-known that the existence of PSD in a patient with progressive dementia is rather suggestive of CJD. However, because of the strong association with CJD, PSD may cause diagnostic confusion when they occur in other disorders. Nevertheless, PSD in EEG has been rarely described in DLB. In this case report, we found that the PSD was presented in DLB after admission, but after the therapy of valproate and diazepam, the EEG has not been improved. According to Brenner [10], PSD is defined as periodic complexes occupying at least 50% of a standard 30-minute EEG, in a symmetric, diffuse and synchronous manner (although they may be more prominent in a given region, frequently the anterior regions) and has a periodicity less than 4.0 seconds. However, the strict criteria for the definition of PSD in CJD may possibly suggest the duration of 100–600 ms, the intercomplex interval of 500–2000 ms, which may possibly assist the differentiation [11]. According to Ferna’ndez-Torre [12], the EEG in DLB has different abnormalities in different phases. During the initial phase of DLB, EEG showed a mild slowing of background activity with diffuse 5–7 Hz rhythms, and occasional bilateral bursts of arrhythmic irregular 1.5–2.0 Hz slow waves. During the intermediate phase, EEG revealed a moderate slowing of background activity with diffuse 4–5 Hz rhythms and frequent burst of multifocal triphasic waves. Finally, during the terminal phase, EEG was dominated by the presence of generalized PSD. On one hand, the patient described here fulfilled the clinical criteria for DLB,on the other hand, the changes in EEG were rare but consistent with previous literature.

Alteration of consciousness and unresponsiveness are typical symptoms of NCSE, however, in some cases, visual hallucinations [13], somatic hallucinations [14], psychotic symptom or myoclonic jerks may also be seen occasionally. Late-onset Absence Status Epilepticus can be seen in elderly patient with generalized spike-wave pattern similar to PSD, which may be difficult to be identified from DLB. But , there were still two points helpful to make differential diagnosis: firstly, in the evolution of the DLB, akinetic-rigid syndrome have occured gradually but not in NCSE; secondly, intravenous valproate or diazepam have terminated the NCSE immediately, but not in DLB, such as in our case.

In summary, PSD in EEG may occasionally be recorded in neurodegenerative disorders such as AD, DLB other than CJD or NCSE. The frequency of such findings in DLB was not known, but the previous case reports suggested that it was not common. Hence it should not dissuade clinicians from the diagnosis of DLB where the clinical and neuropsychological findings are consistent with suggested diagnostic criteria for DLB [9]. The pathophysiological basis of PSD in CJD was uncertain, but has been suggested to reflect the loss of inhibitory parvalbumin binding in thalamic neurons [15]. It would be similar neuropathological markers in other neurodegenerative diseases in which periodic discharges are recorded.

In conclusion, our case showed atypical manifestations and rare EEG findings of DLB which may be confused with NCSE or CJD. Thus, the presence of PSD in a patient with abnormal behavioral or cognition may suggest different neurological diseases, but, DLB needs to be considered. The pathophysiology of PSD needs further exploration.

### Consent

Written informed consent was obtained from the patient’s husband for publication of this case report and any accompanying images. A copy of the written consent is available for review by the Editor of this journal.

## Abbreviations

DLB: Dementia with Lewy body; PSD: Periodic synchronous discharges; EEG: Electroencephalogram; NCSE: Nonconvulsive status epilepticus; AD: Alzheimer’s disease; CJD: Creutzfeldt-Jakob disease.

## Competing interests

The authors declared no conflicts of interest with respect to the research, authorship, funding, and/or publication of this article.

## Authors’ contributions

LS participated in the drafting the manuscript. JC collected the clinical data.FNC participated in the design of the case report. ZW participated in its design and coordination and helped to draft the manuscript. YDL conceived of the case and be accountable for the integrity of any part of the work. All authors read and approved the final manuscript.

## Pre-publication history

The pre-publication history for this paper can be accessed here:

http://www.biomedcentral.com/1471-2377/14/112/prepub
